# Impact of the Error Structure on the Design and Analysis of Enzyme Kinetic Models

**DOI:** 10.1007/s12561-022-09347-5

**Published:** 2022-06-09

**Authors:** Elham Yousefi, Werner G. Müller

**Affiliations:** Department of Applied Statistics, Johannes Kepler University Linz, Linz, Austria

**Keywords:** Nonlinear regression, Logarithmic transformation, D-optimality, Discrimination experiments, D-efficiency, Exact designs

## Abstract

The statistical analysis of enzyme kinetic reactions usually involves models of the response functions which are well defined on the basis of Michaelis–Menten type equations. The error structure, however, is often without good reason assumed as additive Gaussian noise. This simple assumption may lead to undesired properties of the analysis, particularly when simulations are involved and consequently negative simulated reaction rates may occur. In this study, we investigate the effect of assuming multiplicative log normal errors instead. While there is typically little impact on the estimates, the experimental designs and their efficiencies are decisively affected, particularly when it comes to model discrimination problems.

## Introduction

The experimental study of enzyme catalyzed reactions can help to provide valuable information for researches of a great range of specializations. Studying the reversible interaction of drugs binding to their target enzyme is of high importance in pharmaceutical research. Also in drug discovery, (visual) inspection of concentration–response plots is important to diagnose non-ideal behavior and determination of $\mathrm{IC}_{50}$ (see Appendix 1). Appropriate mechanistic and/or kinetic models are instrumental in fulfilling those important goals.

In order to validate the proposed models, we need experimental data collection which requires experimental effort reflected in time, allocation of expenses, manpower, and other costly factors. Optimal experimentation on the other hand can help reduce these expenses by providing high informative data according to the purpose of the experiment. Further, if the theory suggests more than one model, again optimal experimental design plays an important role to provide informative data for discrimination and/or model selection.

Here, as candidate models, we focus on enzyme kinetic models derived from the Michaelis–Menten model. Products are the result of typical binding of substrates (molecules that an enzyme works with) and enzymes (organic catalysts that significantly speed up the rate of chemical reactions within cells) on the active site of the enzymes. The standard two-parameter Michaelis–Menten model is used to describe this type of reaction

1 $$\begin{aligned} E[y]=\dfrac{\theta _\mathrm{V}x_\mathrm{S}}{\theta _\mathrm{M} + x_\mathrm{S} } ~~~~ x_\mathrm{S}\in [a,b] , ~ a\ge 0, \end{aligned}$$

in which *E*[*y*] is used to denote the expected reaction rate, when no inhibition is present. The design, controllable, or independent variable, $x_\mathrm{S}$, represents the substrate concentration supposed to be non-negative due to existence of an initial value to start the enzymatic reactions; e.g., $x_\mathrm{S}\ge 0$. The parameter $\theta _\mathrm{V}$ is the maximum velocity, the system could reach, which should also be non-negatively varying according to physical definition of velocity and $\theta _\mathrm{M}$ is the Michaelis–Menten constant, the value of $x_\mathrm{S}$ at which the maximum velocity is half [1]. Note that according to the mentioned biochemical definitions for the parameters, the expected reaction rate of the system should be greater than or equal to zero. For an overview of optimal design methods with emphasis on applications in Michaelis–Menten models, one may refer to [2].

In some kinetic profiles, the Michaelis–Menten model is extended to include a second controllable variable $x_\mathrm{I}$, i.e., the inhibition concentration (a substance which may cause a reduction in the rate of an enzyme catalyzed reaction) which is taken to be more than or equal to zero in a controlled experiment. Two of such reaction rate equations are competitive and non-competitive inhibition models which are widely used in drug discovery [3] and have already been investigated by many authors in optimal design (see, for example, [4–6]).

*Competitive inhibition* In this type of enzyme catalyzed reaction, the inhibitor competes with the substrate for binding to the pool of free enzyme molecules. Hence binding of an inhibitor to the active site of an enzyme prevents the substrate binding and therefore no product is produced. In this case, the statistical model is

2 $$\begin{aligned} y=\eta _\mathrm{C}+\epsilon =\dfrac{\theta _\mathrm{V}x_\mathrm{S}}{\theta _\mathrm{M}\left( 1+\frac{x_\mathrm{I}}{\theta _\mathrm{K}}\right) + x_\mathrm{S} } + \epsilon , \end{aligned}$$

where $\eta _\mathrm{C}$ denotes the expected reaction rate of the system. $\theta _\mathrm{K}\ge 0$ is the inhibition constant, the concentration required to produce half-maximum inhibition. The independent random errors are normally distributed $\epsilon \sim {\mathcal {N}}(0,\sigma ^2)$. The term statistical model is used instead of the model itself, since in practical studies, observations are exposed to uncontrolled factors like random errors. Therefore, they are included in statistical models here.

*Non-competitive inhibition* This type of inhibition models a system where the inhibitor and the substrate are both bound to the enzyme and form a complex in such a way that the enzyme is inactivated to form a product. The statistical model is defined as

3 $$\begin{aligned} y=\eta _\mathrm{N}+\epsilon =\dfrac{\theta _\mathrm{V}x_\mathrm{S}}{\left( \theta _\mathrm{M}+x_\mathrm{S} \right) \left( 1+\frac{x_\mathrm{I}}{\theta _\mathrm{K}}\right) } + \epsilon , \end{aligned}$$

where $\eta _\mathrm{N}$ is similarly the representation for the expected reaction rate of the model.

*Encompassing model* Atkinson [7] suggested to combine the three-parameter competitive and non-competitive inhibition models to form a four-parameter encompassing model. This model is similarly represented as

4 $$\begin{aligned} y=\eta _\mathrm{E}+\epsilon =\dfrac{\theta _\mathrm{V}x_\mathrm{S}}{\theta _\mathrm{M}\left( 1+\frac{x_\mathrm{I}}{\theta _\mathrm{K}}\right) + x_\mathrm{S}\left( 1+\frac{(1-\lambda )x_\mathrm{I}}{\theta _\mathrm{K}}\right) } + \epsilon , \end{aligned}$$

where $\eta _\mathrm{E}$ is the expected reaction rate of the system. $0\le \lambda \le 1$ is a non-negative parameter, where $\lambda =1$ corresponds to competitive (2) and $\lambda =0$ to non-competitive model (3). See Appendix 1 for a discussion on $\mathrm{IC}_{50}$ determination and positivity of parameters.

Atkinson [4] computed *D*-, $D_s$- and *T*-optimal designs (all being optimality criteria for estimation and discrimination which will be described in Sects. 3 and 4) for competitive, non-competitive inhibition and the encompassing models. The same setting was used in [6] to illustrate their genuinely symmetric discriminating design criterion, called $\delta$-optimality, based on linearization of the models and notion of flexible nominal sets. However, according to biological definitions of parameters and the design variables, the modeled reaction rate needs to be positive, which is not necessarily the case for the additive normal error models used so far. To ensure non-negative values, we suggest instead working with logarithms of the models which assumes multiplicative log normal errors and investigate its effect on estimates and optimal designs.

Enzyme kinetics is a frequent application field in the experimental design literature and Michaelis–Menten-based models have become showcase examples, with recent references abound [8–10] and [11]. While those papers are concentrating on optimal design for parameter estimation, the present work adds to the literature by discussing the model transformation issue in deep. This aspect is also touched as a side issue in the recent paper by [12] but only for parameter estimation, while we also put a focus on model discrimination. That the latter is an important issue for the models introduced above has, for instance, been already discussed in [13].

The rest of the paper proceeds as follows. In Sect. 2, initial parameter estimation for further use is conducted using some real observations. Section 3 provides calculation of optimal designs for precise estimation of the parameters in both the original and the log-transformed models. Next, optimal discriminating designs are derived by making use of compound *T* (*CT*), $D_s$ and $\delta$ criteria. Discriminating performance of all exact optimal designs are compared with each other through a simulation study and contrasted to the results from the additive error case. Discussion on the results plus an interpretation, in terms of pharmacology, for one suggested optimal design is provided in the conclusions.

## Statistical Specification and Estimation

*A standard statistical model* All three models (2), (3), and (4) above could be formulated in terms of a general nonlinear statistical model of *N* observations, as

5 $$\begin{aligned} y_i=\eta (\varvec{\theta },{\mathbf {x}}_i)+\epsilon _i, \quad \quad i=1,\dots ,N, \end{aligned}$$

where $\varvec{\theta }=(\theta _{1},\dots ,\theta _\mathrm{m})^{T}$ is the vector of *m* unknown parameters, $\varvec{\theta }\in \varvec{\Theta }\subseteq {{\mathbb {R}}_{+}^{m}}$, $\varvec{\Theta }$ is a compact set of all non-negative admissible parameter values. ${\mathbf {x}}_i=(x_\mathrm{Si},x_\mathrm{Ii})^{T}$ is the *i*th pair value of design variables. ${\mathfrak {X}}=\left[ [x_\mathrm{S}]_{\min },[x_\mathrm{S}]_{\max }\right] \times \left[ [x_\mathrm{I}]_{\min },[x_\mathrm{I}]_{\max }\right]$ represents the rectangular design region where $0\le [x_\mathrm{S}]_{\min } < [x_\mathrm{S}]_{\max }$ and $0\le [x_\mathrm{I}]_{\min } < [x_\mathrm{I}]_{\max }$ (we may need to discretize the design region for computational purposes). Further, $y_i$ denotes the *i*th observation and $\eta (\varvec{\theta },{\mathbf {x}}_i)$ is the expected response for the *i*th observation, where $\eta : \varvec{\Theta }\times {\mathfrak {X}} \rightarrow {\mathbb {R}}$ is a nonlinear function of the unknown parameters and the design variables.

As briefly noted in Sect. 1, following to the biochemical definitions for the parameters and the pair of design variables ${\mathbf {x}}=(x_\mathrm{S},x_\mathrm{I})^{T}$, the reaction rate *y* in all the above enzyme kinetic models should of course not be negative. This important issue is usually not taken into account by the common practice of simply assuming additive normal errors. It is evident that such errors could potentially lead to negative observations, if their variance is just large enough. Note that negativity of the reaction rate renders the likelihood estimation invalid. Harman and Müller [6] investigated the case to assume multiplicative log normal errors instead to have liberty in inflating the error variance by any factor without producing faulty observations (e.g., for simulation purposes). Now, we suggest to take the natural logarithms of the enzyme kinetics models assuming multiplicative log errors. This way the errors are switched into additive normal and this process is fully matched with the assumptions under which the standard model is defined. Thus, we defined the log model as

6 $$\begin{aligned} \ln (y_i)=\ln (\eta (\varvec{\theta },{\mathbf {x}}_i)\times \epsilon _i)=\ln (\eta (\varvec{\theta },{\mathbf {x}}_i))+\ln (\epsilon _i), \quad \quad i=1,\dots ,N \end{aligned}$$

where $\ln (\epsilon )\sim {\mathcal {N}}(0,\sigma ^2)$. Note that this is a formulation, which has been used in this context before, see, e.g. [14, 15]. Askelöf et al. [16] discusses the related question of the variance of the reaction rate being dependent on the observed velocity.

Therefore, we consider how the designs may differ under the assumption of the log models for enzyme kinetics, compared to their standard ones using both estimation and discrimination criteria. The aim of this research is to investigate how the log models of enzyme kinetics and their error structure may influence the optimal design points produced.

To proceed further with optimal design for a nonlinear model, we usually require some nominal values (see [17]), ideally estimated from data of previous experiments. For such computations in the models (2), (3), and (4), we used data from [5] which consist of $N=120$ triple values of 15 different substrate concentrations (sertraline) spanning a range of [0, 30] while being more dense in lower and more sparse in higher concentrations to provide reasonable substrate saturation as typically is used in [3] and 8 different inhibitor concentration (dextromethorphan) spanning a range of [0, 60] and the reaction rate *y* for each combination of them, resulted from an initial experiment on dextromethorphan–sertraline. Note that the sample size *N* represents the number of observations. All computations were performed using the function nls in R. Besides standard nonlinear least squares estimation, which is used in this paper, there are various other methods to determine the parameters of a Michaelis–Menten model; e.g., [18] uses genetic algorithms or particle swarm optimization, while [19] proposes a Bayesian approach using MCMC techniques. .

The data contained some zero values of substrate concentrations and *y*, which cannot be log-transformed. We have thus replaced these few zeros by some arbitrary small value $\varepsilon$. For a small enough $\varepsilon$ , there is no impact on the estimates in the original model and we have eventually chosen $\varepsilon =0.02$, which renders the smallest possible residual standard error in a back-transformed model (4) (0.1870) compared to the same value in the standard case (0.1526) (see Table 1 ). The residual standard error equations for three different cases are

$$\begin{aligned} \begin{array}{lr} \text{ SSE }=\sum _{i=1}^{N} \left( y_i-\widehat{y_i} \right) ^2, \text{ MSE }=\dfrac{\text{ SSE }}{N-{m}} &{}\text {(The standard case, model (5)) } \\ \text{ SSE}_\mathrm{l}^{*}=\sum _{i=1}^{N} \left( \ln (y_i)-\widehat{\ln ({y}_{i})} \right) ^2, \text{ MSE}_\mathrm{l}^{*} = \dfrac{\text{ SSE}_\mathrm{l}^{*}}{N-{m}} &{}\text {(The log case, model (6))} \\ \text{ SSE}_\mathrm{b}^{*}=\sum _{i=1}^{N} \left( y_i-\exp (\widehat{\ln ({y}_{i})}) \right) ^2, \text{ MSE}_{b}^{*} =\dfrac{\text{ SSE}_{b}^{*}}{N-m} &{}\text {(The back-transformed case)} \end{array} \end{aligned}$$

**Fig. 1:**
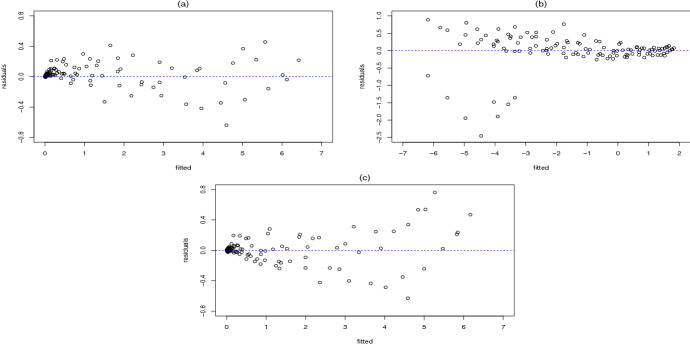
Scatter plot of residuals versus fitted values for **a** standard case, **b** log case, **c** back-transformed case

Here, $N-m$ is the degree of freedom of the corresponding $\text{ SSE }$. The scatter plot of residuals versus fitted values for these cases is displayed in Fig. 1. From the panels a and c, the similarity of the fits is confirmed. Although the residual pattern for the standard case is a bit superior to the one for the back-transformed case from the perspective of being more spread around zero, the advantage of not violating non-negativity motivates us to proceed further with the log model. A robustness analysis was also performed, particularly on the 8 observations in the lower left part of panel b which seem not to follow the trend. It was observed that their deletion would not have any noticeable effect on the initial estimates.

Tables 1 and 2 represent the initial estimations for competitive, non-competitive, and the encompassing models, respectively, in both the standard and the log case. As it is observed, logarithmic transformations do not change the estimates considerably except for $\theta _\mathrm{K}$ in the non-competitive model. Note that there are (slight) discrepancies between the estimates given here and to what Atkinson [4] used for his comparisons. For inner consistency, we decided to only use the values from Tables 1 and 2 throughout this paper.

**Table 1:** The parameter estimates and their corresponding standard error estimates

Competitive model (2)						Non-competitive model (3)					
Standard case (${\hat{\sigma }}=0.1553$)			Log case (${\hat{\sigma }}=0.5160$)			Standard case (${\hat{\sigma }}=0.2272$)			Log case (${\hat{\sigma }}=0.5306$)		
	Estimate ${\hat{\varvec{\theta }}}$	SE ${\hat{\varvec{\sigma }}}$		Estimate ${\hat{\varvec{\theta }}}$	SE ${\hat{\varvec{\sigma }}}$		Estimate ${\hat{\varvec{\theta }}}$	SE ${\hat{\varvec{\sigma }}}$		Estimate ${\hat{\varvec{\theta }}}$	SE ${\hat{\varvec{\sigma }}}$
$\theta _\mathrm{V}$	7.2976	0.1143	$\theta _\mathrm{V}$	6.0645	0.9260	$\theta _\mathrm{V}$	8.6957	0.2227	$\theta _\mathrm{V}$	12.0125	2.0553
$\theta _\mathrm{M}$	4.3860	0.2333	$\theta _\mathrm{M}$	3.2799	0.7288	$\theta _\mathrm{M}$	8.0664	0.4880	$\theta _\mathrm{M}$	8.5359	1.5721
$\theta _\mathrm{K}$	2.5821	0.1454	$\theta _\mathrm{K}$	3.3153	0.6041	$\theta _\mathrm{K}$	12.0566	0.6709	$\theta _\mathrm{K}$	5.6638	0.8879

**Table 2:** The parameter and their corresponding standard error estimates for model (4)

Standard case (${\hat{\sigma }}=0.1526$)			Log case (${\hat{\sigma }}=0.5128$)		
	Estimate ${\hat{\varvec{\theta }}}$	SE ${\hat{\varvec{\sigma }}}$		Estimate ${\hat{\varvec{\theta }}}$	SE ${\hat{\varvec{\sigma }}}$
$\theta _\mathrm{V}$	7.4253	0.1298	$\theta _\mathrm{V}$	6.9897	1.3406
$\theta _\mathrm{M}$	4.6808	0.2724	$\theta _\mathrm{M}$	3.9799	1.0403
$\theta _\mathrm{K}$	3.0581	0.2815	$\theta _\mathrm{K}$	3.7380	0.7218
$\lambda$	0.9636	0.0191	$\lambda$	0.8737	0.1123

Competitive and non-competitive inhibition models are two distinct models, none of which could be obtained from the other by implementing some restrictions on the parameters or through a limiting process. Therefore, in context of Cox’s definition of models to do hypothesis testing [20, 21], these models are (separate) non-nested, although the encompassing model (4) may be used further to ease specification of methods. Similar point is mentioned in [3] Chapter 5, to do some tests for validation of models.

## Optimal Designs for Estimation of Parameters

In this section, we implement *D* and $D_s$ optimality criteria, for model (6) in general. A thorough comparison of the resulted designs in standard and log cases is done using relative efficiency.

*D*-optimal designs, introduced by Wald [22], are used when estimation of all parameters is of primary interest. A design is a set of *n* mutually distinct design points, ${\mathbf {x}}_1,{\mathbf {x}}_2,\dots ,{\mathbf {x}}_n$, with their corresponding proportion of replication of observations, taken at each, (weights) denoted by $\omega _1,\omega _2,\dots , \omega _{n}$ which define a probability measure as $\xi =\left\{ ({\mathbf {x}}_1{^T},\omega _1),({\mathbf {x}}_2{^T},\omega _2),\dots , ({\mathbf {x}}_n{^T},\omega _n)\right\}$, on design region ${\mathfrak {X}}$ such that $\sum _{i=1}^{n} \omega _i=1$. To obtain exact designs, $N_i=N\times \omega _i$ are rounded to integers such that $N=\sum _{i=1}^{n} N_i$ . By an optimal design, we mean a selection of $\xi ^{*}$ which renders an optimum value of some optimality criteria. In the context of enzyme kinetic models, the aim is optimal selection of pairs of substrates and inhibitors in each of the enzyme kinetic models also for their log-transformed cases instead of screening experiments with quite large spans of substrate-inhibition titrations (with 96-, 384-, or 1536-microwell plates being the typical ones, cf. [3]).

The information provided in a design $\xi$ is measured by its Fisher information matrix (FIM), defined in Eq. (8) [23]. Due to dependence of the FIM on the unknown parameters, an initial estimate of them is needed to obtain the locally optimal designs [17]. Consequently, the linearized model at its initial estimate, ${\bar{\varvec{\theta }}}$, is

7 $$\begin{aligned} f({\mathbf {x}}_i,{\bar{\varvec{\theta }}})=\dfrac{\partial \ln (\eta (\varvec{\theta },{\mathbf {x}}_i))}{\partial \varvec{\theta }}\big |_{{\bar{\varvec{\theta }}}} , \end{aligned}$$

where $f^{T}({\mathbf {x}}_i,{\bar{\varvec{\theta }}})$ is the *m* dimensional vector of partial derivatives for the *i*th design point. So the Fisher information matrix for a design with *n* support points is

8 $$\begin{aligned} M(\xi ,{\bar{\varvec{\theta }}})=\sum _{i=1}^{n}\omega _i f({\mathbf {x}}_i,{\bar{\varvec{\theta }}})f^{T}({\mathbf {x}}_i,{\bar{\varvec{\theta }}})={\mathbf {F}}^{T}({\mathbf {X}},{\bar{\varvec{\theta }}}){\mathbf {W}}{\mathbf {F}}({\mathbf {X}},{\bar{\varvec{\theta }}}), \end{aligned}$$

where ${\mathbf {X}}$ denotes the collection of *n* design points. ${\mathbf {F}}({\mathbf {X}},{\bar{\varvec{\theta }}})$ is the $n\times m$ dimensional matrix of partial derivatives and ${\mathbf {W}}$ is the diagonal matrix of weights $\omega _i$.

Optimal designs for estimation of parameters are aimed to maximize a function $\Phi$ of the Fisher information matrix. For the case of D-optimality, the criterion is defined as

9 $$\begin{aligned} \Phi _\mathrm{D}(\xi ,{\bar{\varvec{\theta }}})=\det \left\{ M(\xi ,{\bar{\varvec{\theta }}})\right\} . \end{aligned}$$

Thus, a design is called *D*-optimal, if it maximize the determinant of the information matrix (or similarly if it minimize the determinant of the covariance matrix). In order to compare any design to a *D*-optimum one, we used *D*-efficiency defined as

10 $$\begin{aligned} \text {Eff}_\mathrm{D}(\xi )=\left[ \dfrac{\det \left\{ M(\xi ,{\bar{\varvec{\theta }}}\right\} }{\det \left\{ M(\xi ^{*},{\bar{\varvec{\theta }}})\right\} }\right] ^{\frac{1}{m}}. \end{aligned}$$

When a design $\xi$ with *N* observations and $\text {Eff}_\mathrm{D}$ is used in an experiment, the same accuracy in estimation is achieved by performing only $N\times \text {Eff}_\mathrm{D}$ trials under the optimal design $\xi ^{*}$. Note that all applied equivalence theorems are given in Appendix 3.

Note that for nonlinear models, *D*-optimality is only suitable when the so-called parameter curvature is negligible. Hamilton & Watts [24] proposed to instead consider a quadratic design criterion based on second-order approximation of the volume of the parameter inference region, when the sample size is small. To investigate this effect, we computed this criterion for the encompassing model in both the standard and the log cases. It is observed that this effect is actually negligible and the new designs based on the proposed criterion are essentially the same as the *D*-optimum ones for the encompassing model in Table 3, with minor deviations as seen from Table 8 of Appendix 2.

$D_s$-optimality, introduced by Atkinson & Cox [25], is a special case of *D*-optimality, when the interest is in estimation of a subset of *s* (which is equal to one in our case) parameters while the other $m-s$ parameters can be considered being nuisance. For more details on the $D_s$-optimality formulation, one may refer to [23].

Table 3 presents the *D* and $D_s$-optimal designs consisting of recalculations of the designs for the standard case already presented in [4] with the difference that here the design region is the discretized rectangular ${\mathfrak {X}}=[0,30]\times [0,60]$ and initial parameter estimations are taken from Tables 1 and 2 then followed by optimal design calculations for the log case. For $D_s$-optimality, we assume that $s=1$ meaning that we are interested in computation of optimal designs for estimation of a single parameter of interest, $\lambda$, in the encompassing model such that a precise estimation of $\lambda$ test whether a simpler model is adequate. The design region used for the log case is the rectangular ${\mathfrak {X}}=[\varepsilon ,30]\times [0,60]$ constructing a grid of $31\times 61$ points (note that a denser grid of the points does not affect the final resulted designs in all considered criteria of optimality in the log case due to accumulation of optimal support points solely on the corners of the design region, being described later, and therefore speeds up the calculations). Assumed parameter spaces can be $\varvec{\theta }\in \left( 0,\infty \right)$, but sometimes for computational purposes, we had to use nonrestrictive upper bounds. Note that some discrepancies in the design recalculations of the standard case compared to the designs presented in [4] are due to differences in the initial estimates and the designs space. Note that for computation of all *D*-optimal designs, we used the package OptimalDesign in R and a linear programming simplex method [26] was used for computation of $D_s$ optimal designs.

**Table 3:** *D* and *Ds*-optimal designs

	Design	$x_\mathrm{S}$	$x_\mathrm{I}$	$\omega$
Standard case	$4D_\mathrm{N}$	30.000	0.000	0.25
		5.223	0.000	0.25
		30.000	12.045	0.25
		5.223	12.045	0.25
	$4D_\mathrm{C}$	30.000	0.000	0.25
		3.348	0.000	0.25
		30.000	20.297	0.25
		7.902	7.137	0.25
	$4D_\mathrm{E}$	30.000	0.000	0.25
		3.616	0.000	0.25
		30.000	18.290	0.25
		7.500	7.584	0.25
	$Ds_\mathrm{N}$	30.000	0.000	0.086
		3.884	0.000	0.208
		30.000	16.952	0.206
		3.884	16.952	0.500
	$Ds_\mathrm{C}$	30.000	0.000	0.027
		2.545	0.000	0.088
		30.000	28.550	0.371
		7.098	8.253	0.514
Log case	$4D_\mathrm{N},3D_\mathrm{N},4D_\mathrm{C},4D_\mathrm{E},Ds_\mathrm{N}$	$\varepsilon$	0	0.25
		30	0	0.25
		$\varepsilon$	60	0.25
		30	60	0.25
	$3D_\mathrm{C}$	$\varepsilon$	0	1/3
		30	0	1/3
		$\varepsilon$	60	1/3
	$Ds_\mathrm{C}$	$\varepsilon$	0	0.017
		30	0	0.173
		$\varepsilon$	60	0.327
		30	60	0.483

It is remarkable that all the optimal designs for the log model are concentrated at the corners of the design region with the interpretation that the best designs for precise estimation of parameters are the most extreme pair concentrations of substrate and inhibition which makes them easy to use in practice. Also, they are robust to the choice of initial estimates, which indicate that they behave much like linear models over a wide region of the parameter space, another attractive feature. Note that $4D_\mathrm{N}$ and $4D_\mathrm{C}$ stand for *D*-optimal designs when the last parameter of the encompassing model is equal to $\lambda =0$ or $\lambda =1$, respectively, using the initial estimates for the non-competitive and competitive models in Table 1. $4D_\mathrm{E}$ is the *D*-optimal design for the four-parameter encompassing model using the initial estimates in Table 2. Further, $3D_\mathrm{N}$ denotes the *D*-optimal design for the three-parameter non-competitive model, which surprisingly has four points of support and $3D_\mathrm{C}$ is similarly computed for the competitive model. $Ds_\mathrm{N}$ and $Ds_\mathrm{C}$ are $D_s$-optimum for estimation of $\lambda$ in the encompassing model when $\lambda =0$ and $\lambda =1$, respectively. Similar recalculations of designs for the standard case shows that in all these cases, optimal designs are more spread over the rectangular design region and not completely located in the extremes. Note that in the standard case, omitted from the table, $3D_\mathrm{N}$ and $3D_\mathrm{C}$ are the first three support points of their corresponding designs $4D_\mathrm{N}$ and $4D_\mathrm{C}$ with weights of 1/3 each. The difference in optimal resulting designs in both the log and standard cases once again highlights the importance to know which error structure to use in an experiment.

This is even emphasized by looking at Table 4, which presents a comprehensive comparison of all the *D* and $D_s$ designs using relative *D* and $D_s$ efficiencies. The upper parts of the table are the efficiencies of all designs relative to the designs of the standard case, whereas the lower parts are relative to the log case designs. We use the symbol of – to indicate that due to not having enough support points, the information matrices are not full rank and therefore the designs are singular. The following conclusions may be drawn from the table:

- Naturally, higher efficiencies are observed whenever similar cases are relatively compared; i.e., when designs of the standard case are relative to designs of the standard case or the designs of the log case are relative to the log case designs.
- For the case of the standard model considered as the reference (i.e., the model in the denominator of relative efficiency), we typically see that the efficiencies are always higher when the designs are compared to the encompassing rather than the pure models (except for $Ds_\mathrm{C}$ in the standard and log case compared to $4D_\mathrm{C}$ and $3D_\mathrm{C}$ in the lower part). For example, notice the *D*-efficiencies 100 (38.26) and 87.01 (52.70) in the first row of the table. The situation is exactly reverse for the log case.
- Smaller efficiencies are observed when the log case designs are relative to standard designs and the other way around. Higher defects are observed in designs of the log case relative to the standard case designs. Notice the values in the last three rows of the upper part of the table with the first seven rows of the lower part.

The latter observation indicates that while the log case designs are robust to misspecification of nominal values, they are much less so for misspecification of the error structure. It seems when an experimenter is unsure about that it is much safer to use the additive normal error specification.

To check if designs of the log case in Table 3 are actually optimum, we have eventually employed the respective equivalence theorems. Their sensitivity functions, Eq. (21), are plotted in Fig. 4 in Appendix 4, which shows the same maximal value (equal to the number of their respective parameters) at the points of supports of the optimal designs.

**Table 4:** The *D* and *Ds* efficiencies for all *D* and *Ds* designs

	Design	Standard case, reference model						
		Eff$_\mathrm{D}(\%)$					Eff$_\mathrm{Ds}(\%)$	
		$4D_\mathrm{N}$	$3D_\mathrm{N}$	$4D_\mathrm{C}$	$3D_\mathrm{C}$	$4D_\mathrm{E}$	$Ds_\mathrm{N}$	$Ds_\mathrm{C}$
Standard case	$4D_\mathrm{N}$	100	87.01	85.12	76.95	88.58	72.19	47.37
	$3D_\mathrm{N}$	−	100	−	91.76	−	−	−
	$4D_\mathrm{C}$	87.36	83.30	100	87.55	99.67	47.91	61.25
	$3D_\mathrm{C}$	−	91.41	−	100	−	−	−
	$4D_\mathrm{E}$	90.74	85.13	99.69	87.04	100	52.25	61.57
	$Ds_\mathrm{N}$	78.04	56.08	63.67	51.63	66.46	100	49.10
	$Ds_\mathrm{C}$	47.35	37.37	56.39	34.64	55.85	45.78	100
Log case	$4D_\mathrm{N},3D_\mathrm{N},4D_\mathrm{C},4D_\mathrm{E},Ds_\mathrm{N}$	0.70	2.76	0.49	4.69	0.50	0.01	0.00
	$3D_\mathrm{C}$	−	0.08	−	0.07	−	−	−
	$Ds_\mathrm{C}$	0.41	1.42	0.29	2.11	0.30	0.00	0.00

	Design	Log case, reference model						
		Eff$_\mathrm{D}(\%)$					Eff$_\mathrm{Ds}(\%)$	
		$4D_\mathrm{N}$	$3D_\mathrm{N}$	$4D_\mathrm{C}$	$3D_\mathrm{C}$	$4D_\mathrm{E}$	$Ds_\mathrm{N}$	$Ds_\mathrm{C}$
Standard case	$4D_\mathrm{N}$	38.26	52.70	25.92	30.84	30.08	14.63	6.96
	$3D_\mathrm{N}$	−	44.26	−	19.97	−	−	−
	$4D_\mathrm{C}$	35.81	52.82	27.98	30.88	30.86	11.15	9.42
	$3D_\mathrm{C}$	−	56.22	−	31.38	−	−	−
	$4D_\mathrm{E}$	36.04	52.32	27.43	30.33	30.56	11.78	9.18
	$Ds_\mathrm{N}$	40.80	55.00	30.47	35.89	34.22	16.65	8.44
	$Ds_\mathrm{C}$	24.43	31.82	21.20	17.57	22.48	11.05	16.85
Log case	$4D_\mathrm{N},3D_\mathrm{N},4D_\mathrm{C},4D_\mathrm{E},Ds_\mathrm{N}$	100	100	100	87.50	100	100	67.55
	$3D_\mathrm{C}$	−	83.99	−	100	−	−	−
	$Ds_\mathrm{C}$	58.72	80.32	58.72	37.75	58.72	22.94	100

Note: − Singular designs

## Optimal Designs for Model Discrimination

We have investigated optimal designs for parameter estimation of each model. If the models are more than one (like in our case) and there is uncertainty in which model to choose, we need to perform experiments to find optimal discriminating designs. Note that the $D_s$-optimality can be used for model discrimination as well. As the encompassing model discriminates the competitive and the non-competitive model by the respective value of the parameter $\lambda$, it is natural that good estimation of $\lambda$ ensures good discriminability. However, note that there is actually a great range of possible encompassing model specification and that the chosen one is subject to considerable arbitrariness.

### T-optimal Designs

Another widely used discrimination criterion is *T*-optimality introduced by Atkinson & Fedorov [27] which maximizes the non-centrality parameter of the *F*-test for departures from the wrong model when the assumption is to know the true model and its fixed parameters, ${\bar{\varvec{\theta }}}_0$, so that the resulting optimal design depends on ${\bar{\varvec{\theta }}}_0$ in the assumed true model and therefore will be locally optimum as well. To tackle dependence of the discriminating designs on the true parameter values, we have considered some sequential design procedures. A comparison between their performances and some details on the speed of their convergences are given in [28]. We denote those two models as $\eta _0(\varvec{\theta }_0,{\mathbf {x}})$ and $\eta _1(\varvec{\theta }_1,{\mathbf {x}})$ and the subscripts zero and one are just suggesting the assumed true and wrong models. Therefore, by assuming the first model to be true, a design $\xi _{T0}^{*}$ would be called *T*-optimal if it maximizes the lack of fit sum of squares for the second model as

11 $$\begin{aligned} \Delta _0(\xi )&=\sum _{i=1}^{n}\omega _i\left( \eta _0({\bar{\varvec{\theta }}}_0,{\mathbf {x}}_i)- \eta _1({\hat{\varvec{\theta }}}_1,{\mathbf {x}}_i)\right) ^2 \nonumber \\&=\inf _{\varvec{\theta }_1\in \varvec{\Theta }_1}\sum _{i=1}^{n}\omega _i\left( \eta _0({\bar{\varvec{\theta }}}_0,{\mathbf {x}}_i)- \eta _1(\varvec{\theta }_1,{\mathbf {x}}_i)\right) ^2, \end{aligned}$$

where ${\hat{\varvec{\theta }}}_1$ is the estimate derived from minimization of (11). Let $\Xi$ be a set of all approximate designs. Then, the design $\xi _{T0}^{*}\in \Xi$ will be called *T*-optimal, if

12 $$\begin{aligned} \xi _{T0}^{*}\in \arg \max _{\xi \in \Xi }\Delta _0(\xi ), \end{aligned}$$

In order to compare any design to a *T*-optimum design $\xi _{T0}^{*}$, *T*-efficiency is defined as

13 $$\begin{aligned} \text {Eff}_{T0}(\xi )=\dfrac{\Delta _0(\xi )}{\Delta _0(\xi _{T0}^{*})}. \end{aligned}$$

The same definitions hold when $\eta _1$ is assumed as the true model and only the indices in (11)–(13) are interchanged. Atkinson [4] introduced the Compound *T*- (*CT*-) optimal designs to discriminate between both models which maximize a weighed product of efficiencies as

14 $$\begin{aligned} \left\{ \text {Eff}_{T0}\right\} ^{1-\nu }\left\{ \text {Eff}_{T1}\right\} ^{\nu }=\left\{ \dfrac{\Delta _0(\xi )}{\Delta _0(\xi _{T0}^{*})} \right\} ^{1-\nu }\left\{ \dfrac{\Delta _1(\xi )}{\Delta _1(\xi _{T1}^{*})} \right\} ^{\nu }, 0\le \nu \le 1. \end{aligned}$$

Here $\nu$ is a weighting coefficient such that $\nu =0$ refers to *T*-optimal designs when $\eta _0$ in assumed true and $\nu =1$ for $\eta _1$, similarly. By taking the logarithms of the right-hand side of (14) and omitting the constant values, the *CT*-criterion is

15 $$\begin{aligned} \Phi _\mathrm{CT}(\xi )=(1-\nu )\ln \Delta _0(\xi )+\nu \ln \Delta _1(\xi ), \end{aligned}$$

which is a convex combination of two design criteria, each of which is the logarithm of that for *T*-optimality. Further, *CT*-criterion satisfies the conditions of convex optimum design theory [4]. Atkinson & Fedorov [27] obtained an analogous of *D*-equivalence theorem to provide a check of *T*-optimal designs reproduced in Appendix 3.

Similar to [4], we computed four approximate discriminating designs denoted here by $A_1$–$A_4$ also for the log case, presented in the left-hand part of Table 5. $A_1$ corresponds to a *T*-optimal design when the non-competitive model (3) holds and the nominal parameter values are the estimates in the log case of the right-hand side of Table 1. $A_2$ corresponds to a *CT*-optimal designs for $\nu =0.5$ with the corresponding nominal values in Table 1. $A_3$ is the $D_s$ optimal design for $\lambda$ in model (4) at a nominal value of $\lambda =0.8737$. The estimates of parameters in Table 2 are used as nominal values. The last design $A_4$ refers to a *T*-optimal design when the competitive model (2) holds with the corresponding nominal values in the log case of left-hand side of Table 1. The right-hand part of Table 5 corresponds to recalculations of Atkinson’s designs for the standard case. Again some discrepancies are observed in the optimal designs of the standard case here, compared to the values reported in [4] due to the differences in the nominal parameter values and the design space and accordingly, some differences in the *T*-efficiencies have occurred.

**Table 5:** Some optimal discriminating designs and their *T*-efficiencies

Log case (Eq. 6 )						Standard case (Eq. 5 )					
				Eff$_\mathrm{T} (\%)$						Eff$_\mathrm{T} (\%)$	
Design	$x_\mathrm{S}$	$x_\mathrm{I}$	$\omega$	$A_1$	$A_4$	Design	$x_\mathrm{S}$	$x_\mathrm{I}$	$\omega$	$\nu =0$	$\nu =1$
$A_1$	$\varepsilon$	0	0.0095	100	76.50	$\nu =0$	30.000	0.000	0.063	100	57.40
	30	0	0.1402				3.214	0.000	0.063		
	$\varepsilon$	60	0.3600				30.000	21.413	0.310		
	30	60	0.4903				5.625	11.152	0.564		
$A_2$	$\varepsilon$	0	0.1688	74.89	89.72	$\nu =0.5$	30.000	0.000	0.058	86.52	80.52
	30	0	0.1818				3.348	0.000	0.189		
	$\varepsilon$	60	0.3002				30.000	22.082	0.260		
	30	60	0.3492				5.759	11.375	0.493		
$A_3$	$\varepsilon$	0	0.1633	72.96	92.40	$\lambda =0.9636$	30.000	0.000	0.052	90.37	77.01
	30	0	0.2189				2.678	0.000	0.137		
	$\varepsilon$	60	0.2811				30.000	25.874	0.330		
	30	60	0.3367				6.562	8.922	0.481		
$A_4$	$\varepsilon$	0	0.2500	57.85	100	$\nu =1$	30.000	0.000	0.060	76.93	100
	30	0	0.2502				3.080	0.000	0.250		
	$\varepsilon$	60	0.2500				30.000	22.751	0.250		
	30	60	0.2498				5.491	11.598	0.440		

As we can observe from Table 5, again all the support points of the log case designs are the same and on the corners of the design space with the difference only due to their corresponding weights $\omega$. To check optimality of these designs, see again their sensitivity functions (Eq. (22) for $A_1$, $A_2$ , and $A_4$) plotted in Fig. 5 in Appendix 4. Further, we used the Fedorov-Wynn algorithm ( [23]) to find the optimal designs $A_1$, $A_2$ , and $A_4$ and set the maximum iteration equal to a fixed number (sufficiently large to ensure convergence of designs) as the stopping rule of the algorithm. Efficiencies are relatively high in both the log and standard cases whether we assume $A_1$ or $A_4$ as the reference designs in the denominator of the Eff$_\mathrm{T} (\%)$. So we may state that the product of efficiencies is high for all the designs specifically for $A_2$ and $A_3$ in the log case and $\nu =0.5$ and $\lambda =0.9636$ in the standard case, regardless of which model holds in comparisons, if we exclude the cases which require the assumption of true model. This provides a better interpretation if we are not interested in assuming any of the competitive or non-competitive models to hold.

### $\delta$-optimal Designs

The last discrimination procedure used here is $\delta$-optimality introduced by Harman & Müller [6]. The method is a genuinely symmetric design criterion (no assumption on the true model is required) which is defined to discriminate between two statistical models of the form $\eta _\mathrm{u}(\varvec{\theta }_\mathrm{u},{\mathbf {x}}_i)=\eta _\mathrm{u}$ for $u=0,1$ and $i=1,\dots ,N$ with the same number of parameters *m*. We denote the size of exact designs by *N*, equal to the number of observations, since replications are allowed. The idea of the method is to linearize both models at their respective nominal values, denoted by ${\tilde{\varvec{\theta }}}_\mathrm{u}$, as

$$\begin{aligned} (y_i)_{i=1}^{N}\approx {\mathbf {F}}_\mathrm{u}({\mathcal {D}})\varvec{\theta }_\mathrm{u}+{\mathbf {a}}_\mathrm{u}({\mathcal {D}})+\epsilon ,\quad u=0,1, \end{aligned}$$

where ${\mathcal {D}}=({\mathbf {x}}_1, \dots , {\mathbf {x}}_N)$ is an exact design of size *N* and ${\mathbf {F}}_\mathrm{u}({\mathcal {D}})$ is the $N\times m$ matrix of partial derivatives similarly computed from Eq. (7). Further ${\mathbf {a}}_\mathrm{u}({\mathcal {D}})$ is a vector as

$$\begin{aligned} {\mathbf {a}}_\mathrm{u}({\mathcal {D}})=(\eta _\mathrm{u}({\tilde{\varvec{\theta }}}_\mathrm{u},{\mathbf {x}}_i))_{i=1}^{N}-{\mathbf {F}}_\mathrm{u}({\mathcal {D}}){\tilde{\varvec{\theta }}}_\mathrm{u}. \end{aligned}$$

According to above notations, the linearized distance criterion is (see [6] for more details)

16 $$\begin{aligned}&\delta ({\mathcal {D}})=\inf _{\varvec{\theta }_{0}\in {\tilde{\varvec{\Theta }}}_{0},\varvec{\theta }_{1}\in {\tilde{\varvec{\Theta }}}_{1}}\delta ({\mathcal {D}}\mid \varvec{\theta }_{0},\varvec{\theta }_{1}). \nonumber \\ \delta ({\mathcal {D}}\mid \varvec{\theta }_{0},\varvec{\theta }_{1})&=\Vert {\mathbf {a}}_0({\mathcal {D}})+{\mathbf {F}}_0({\mathcal {D}})\varvec{\theta }_{0}-\left\{ {\mathbf {a}}_1({\mathcal {D}})+{\mathbf {F}}_1({\mathcal {D}})\varvec{\theta }_{1}\right\} \Vert , \end{aligned}$$

where ${\tilde{\varvec{\Theta }}}_{0}\subseteq {\mathbb {R}}^{m},{\tilde{\varvec{\Theta }}}_{1}\subseteq {\mathbb {R}}^{m}$ are called the flexible nominal sets which will not be considered fixed like the parameter spaces $\varvec{\Theta }_{0}$ and $\varvec{\Theta }_{1}$. Further the $\delta$-criterion, defined as a function of the exact design ${\mathcal {D}}$, is represented using the counting measure $\zeta$ on ${\mathfrak {X}}$ as

$$\begin{aligned} {\zeta }\left( \left\{ {\mathbf {x}}\right\} \right) := \# \left\{ i\in \left\{ 1, \dots , N\right\} : {\mathbf {x}}_i={\mathbf {x}}\right\} , {\mathbf {x}}\in {\mathfrak {X}}. \end{aligned}$$

where $\zeta$ here is the collection of exact designs of size *N* with integer replications compared with $\xi$ which refers to probability measures and continuous weights in the approximate case. By construction, the $\delta$-criterion can be interpreted as a minimal distance between the expectation surfaces for the two compared models. More on this and a discussion of its convexity can be found in [6]. Finally for a set ${\mathfrak {D}}$ of all *N*-point designs, a design ${\mathcal {D}}^{*}\in {\mathfrak {D}}$ will be called $\delta$-optimal, if

17 $$\begin{aligned} {\mathcal {D}}^{*}\in \arg \max _{{\mathcal {D}}\in {\mathfrak {D}}}\delta ({\mathcal {D}}) \end{aligned}$$

We need to emphasize that $\delta$-optimal designs are evaluated using the rapid and stable method for bounded variable least squares implemented in R package bvls (see [29] and [30] ). Therefore, for implementation purposes, $\delta ^{2}({\mathcal {D}}\mid \varvec{\theta }_0,\varvec{\theta }_1)$ is used as

18 $$\begin{aligned} \delta ^{2}(D\mid \varvec{\theta }_0,\varvec{\theta }_1)=\Vert \left\{ {\mathbf {a}}_0({\mathcal {D}})-{\mathbf {a}}_1({\mathcal {D}})\right\} -\left[ - {\mathbf {F}}_0({\mathcal {D}}),{\mathbf {F}}_1({\mathcal {D}})\right] \varvec{\theta }\Vert ^{2}, \end{aligned}$$

where $\varvec{\theta }$ is the compound vector of unknown parameter vectors in both models. Besides, in computation of $\delta$-optimal designs, we used the standard KL-exchange heuristic [23]. Starting with a random sample of size *N*, the algorithm keeps making exchanges until no improvement in the criterion value is observed and this suggests resulting in nearly optimal designs. The method requires the choice of nominal values ${\tilde{\varvec{\theta }}}_u={\hat{\varvec{\theta }}}_u$ and nominal intervals ${\tilde{\varvec{\Theta }}}_u=[ {\tilde{\theta }}_{u1}\pm r{\tilde{\sigma }}_{u1}] \times [ {\tilde{\theta }}_{u2}\pm r{\tilde{\sigma }}_{u2}] \times [ {\tilde{\theta }}_{u3}\pm r{\tilde{\sigma }}_{u3}]_{u=0,1}$ in which ${\tilde{\theta }}_{uv}={\hat{\theta }}_{uv}$ and ${\tilde{\sigma }}_{uv}={\hat{\sigma }}_{uv}$ for $u=0,1$ and $v=1,2,3$ (${\hat{\theta }}_{uv}$ are basically the estimates of parameters of the models). Note that $r\ge 0$ works as a tuning parameter which is specialized to change the size of nominal intervals and plays an important role in computation of the $\delta$-optimal designs. Therefore, we denote by $\delta _r$ a $\delta$-optimal design for a specific value of *r*.

Returning to our example, we would like to compute $\delta$-optimal designs for models (2) and (3) in the log case. According to Table 1 for initial estimates of the log cases, $r\in \left\{ 1,2,3,4 \right\}$ higher values of which cause some or all values in the lower bounds of nominal intervals become negative. Therefore, to fulfill this constraint, we used three alternatives to prevent having negative nominal intervals for values of *r* more than $r>4$. The first alternative *a*) was to increase *r* and cut the lower bounds of the nominal intervals at zero wherever they are negative. The second *b*) was to add the absolute values of negative lower bounds of the nominal intervals, cut at zero, into theirs upper bounds (shifting the upper bounds). For the third alternative *c*), we used a method based on reverse transformation.

### A Simulation Study of Discriminating Designs

In this part, we designed two experiments, one in a small and one in a large scale, to compare discriminatory power of all (designs resulted from) methods of this section. Results would guide the experimenters, willing to work with log models of enzyme inhibitions, a path on which discriminating method to choose in applications.

#### Exact Designs, $N=6,7,8,9$

Since the approximate designs of Table 5, resulted from *T*, *CT*, and $D_s$ criteria, have varying weights compared together and therefore they have different number of replications while rounding into exact ones, for comparison with (exact) $\delta$-optimal designs, and also in order to observe how the designs will behave while their size changes, we designed experiments for $N=6,7,8,9$. We computed average values of correct classification (hit) rates when both models contribute equally in simulations presented in Table 6. Note that the first support point of the design $A_1$ in Table 5 will not contain replications for $N=6,7,8,9$ due to its very low weight, $\omega =0.0095$. Also to test discriminatory performance of $\delta$-optimal designs for different values of *r*, the tuning parameter is set to $r=\left\{ 1,2,3,4,5,10,15\right\}$. For this part, we use the error standard deviation estimate equal to ${\hat{\sigma }}=0.5128$ from the encompassing model in the log case as a base value for the simulation error standard deviation.

As we can observe from Table 6, $A_2$, $A_3$ , and $\delta _4$ and more specifically $A_2$ have the best performance for all number of exact designs when both models contribute equally in simulations. This result would be of high importance to those who seek to implement a tested method for discriminating between log models of enzyme inhibition.

**Table 6:** Average values of hit rates (AvHr) for $B=100$ and $N=6$–9

$N=6$		$N=7$		$N=8$		$N=9$	
Designs	AvHr	Designs	AvHr	Designs	AvHr	Designs	AvHr
$A_1,\delta _{1},\delta _{2},\delta _{3}$	70.470	$A_1$	71.980	$A_1,\delta _{2}$	71.200	$A_1$	71.995
$A_2,A_3$	$\mathbf{89.665}$	$A_2,\delta _{4}$	$\mathbf{90.925}$	$A_2,A_3,\delta _{3},\delta _{4}$	$\mathbf{92.595}$	$A_2,A_3$	$\mathbf{93.635}$
$A_4$	87.525	$A_3$	90.675	$A_4$	91.640	$A_4$	92.715
$\delta _4$	87.865	$A_4$	87.365	$\delta _1$	71.275	$\delta _{1},\delta _{2},\delta _{3}$	70.910
$\delta _{5a}$	86.750	$\delta _1,\delta _{2},\delta _{3}$	70.680	$\delta _{5a},\delta _{5b}$	91.375	$\delta _{4}$	93.230
$\delta _{5b},\delta _{5c}$	88.430	$\delta _{5a}$	89.470	$\delta _{5c}$	90.585	$\delta _{5a},\delta _{5b}$	91.870
$\delta _{10a}$	75.590	$\delta _{5b},\delta _{5c}$	89.690	$\delta _{10a}$	78.710	$\delta _{5c}$	91.115
$\delta _{10b}$	74.685	$\delta _{10a}$	78.185	$\delta _{10b},\delta _{15a},\delta _{15b}$	77.130	$\delta _{10a}$	78.935
$\delta _{10c}$	79.065	$\delta _{10b}$	76.985	$\delta _{10c}$	80.070	$\delta _{10b},\delta _{15a},\delta _{15b},\delta _{15c}$	78.995
$\delta _{15a}$	73.430	$\delta _{10c}$	79.005	$\delta _{15c}$	81.530	$\delta _{10c}$	83.825
$\delta _{15b}$	72.725	$\delta _{15a},\delta _{15b},\delta _{15c}$	75.020				
$\delta _{15c}$	73.555						

#### A Large-Scale Experiment, $N=60$

In the second part of simulations, we designed an experiment to compute total correct classification rates and also the average classification rates of all designs for $N=60$ . Since the discriminatory power of all the designs for $N=60$ is perfect and rather the same when the estimated error standard deviation, ${\hat{\sigma }}=0.5128$, is used we are required to inflate it. Therefore, the error standard deviation used is $4\times {\hat{\sigma }}$. The number of Monte Carlo simulations done is $B=1000$. We need to mention that here, the tuning parameter is set to contain also $r=6$, beside the values used for the last part of the low-scale experiment.

**Fig. 2:**
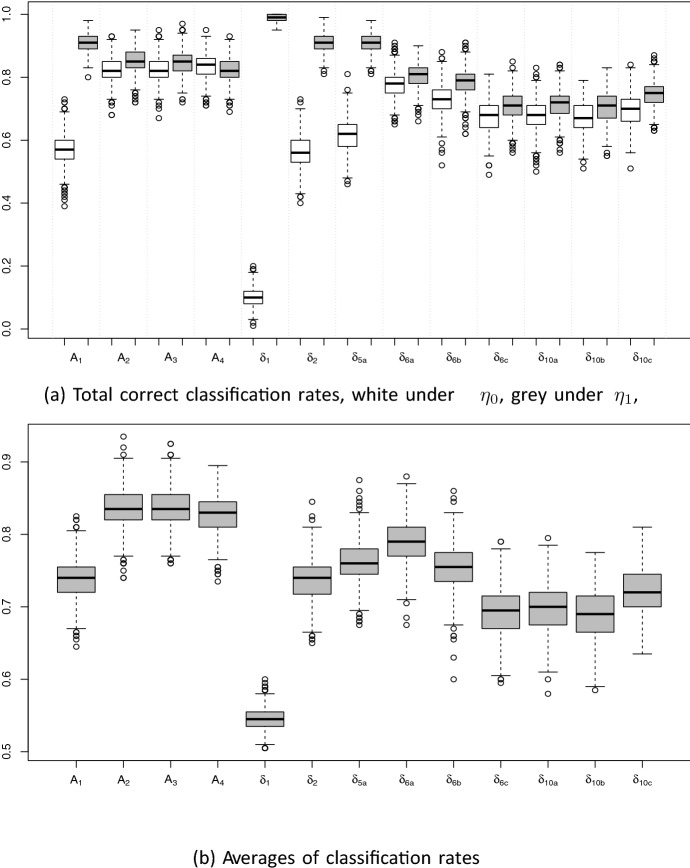
Boxplots for the correct classification rates of all designs $r=\left\{ 1,2,3,4,5,6,10,15 \right\}$, $\delta _{2}$ stands for all: $\delta _{2},\delta _{3},\delta _{4},\delta _{5b},\delta _{5c},$ and $\delta _{10b}$ stands for all: $\delta _{10b},\delta _{15a},\delta _{15b},\delta _{15c}$.

The corresponding box plots of the total and average correct classification rates are given in Fig. 2. All designs have reasonably high performances except the design $\delta _{1}$. Designs $A_2$ and $A_3$ and to be more specific $A_2$ is performing fairly well according to both panels in Fig. 2a and b which confirm the results presented in Table 6. Note that $A_1$ and $A_4$ are excluded from our comparisons, since the methods they are resulted from are inherently asymmetric. Among the designs resulting from the symmetric method $\delta$-optimality, $\delta _{6a}$ is also performing well suggesting that $r=6$ is a good choice for the tuning parameter.

## Conclusions

This paper provided optimal designs with high efficiencies either for estimation of the parameters of interest or for discriminating enzyme kinetics log models whichever model holds. One should be careful which error structure to choose since the resulting designs showed considerably differing patterns. In the standard case, optimal designs are spread over the design region while in the log case, they are typically concentrated on its corners. This means optimal choices of the designs should contain the most extreme pair concentrations of the substrates and inhibitors with different replications, and therefore, it is important to be aware of how to choose them in the experiment. Misspecification of those concentrations may lead to irrecoverable results in producing dextromethorphan–sertraline and other similar biochemical products. There is no such sensitivity on how to choose the design region in the standard case as the designs are typically not dependent upon where the boundaries are set. On the other hand, those designs are much more sensitive to the nominal values chosen, which is not the case for the log-transformed model.

Both these observations for the log model are in accordance with the behavior of linear models. So while those transformed models are not intrinsically linear cf. [31], this still points to the suspicion that their curvature must be flat for a wide range in the parameter space. The resulting robustifying effect on the designs may then be a desired quality for the experimenter.

One other interesting result is that the optimal designs for discriminating between the nonlinear log models are similar to optimal designs for precise estimation of parameters of each model, in all but one case, with the only difference in their corresponding weights.

Finally, it was observed that in such transformed models - despite a firm theoretical grounding - both $A_2$ and $A_3$ provided high relative efficiencies (or product of relative efficiencies), when the interest is to solely compare design methods avoiding asymmetries. In particular, since we are more concerned with real discriminatory power in practical situations, comparisons are more straightforward using the results from the Sect. 4.3. In particular, $A_2$ and $A_3$ have the best performances according to high average values of hit rates in Table 6. They also perform well in parameter estimation as their D-efficiencies (in the encompassing model) are above $95\%$. We note that since $D_s$-optimal designs are easy to calculate in comparison with *CT* or $\delta$-optimal designs, this makes the design $A_3$ particularly attractive. If there is no such constraint about the method complexity or the time required for calculations, $A_2$ resulted from the *CT*-optimal design criterion would also be a recommendable choice (best choice when compared to the competitors in the present manuscript) for discriminating log models of enzyme inhibition due to gaining the highest average rates of classification while still being sufficiently efficient for parameter estimation.

**Fig. 3:**
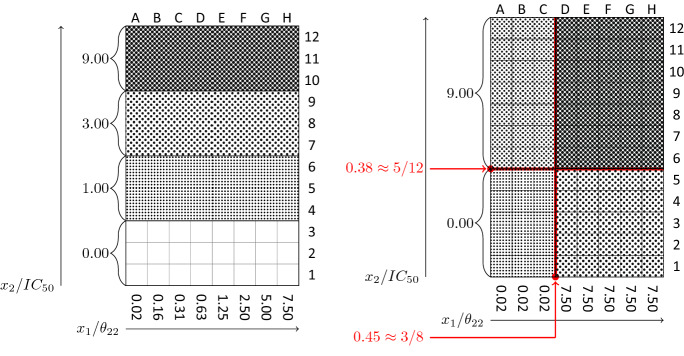
96-well plate format for inhibitor modality studies. left: usual format, right: adapted for $A_3$ optimal design

**Fig. 4:**
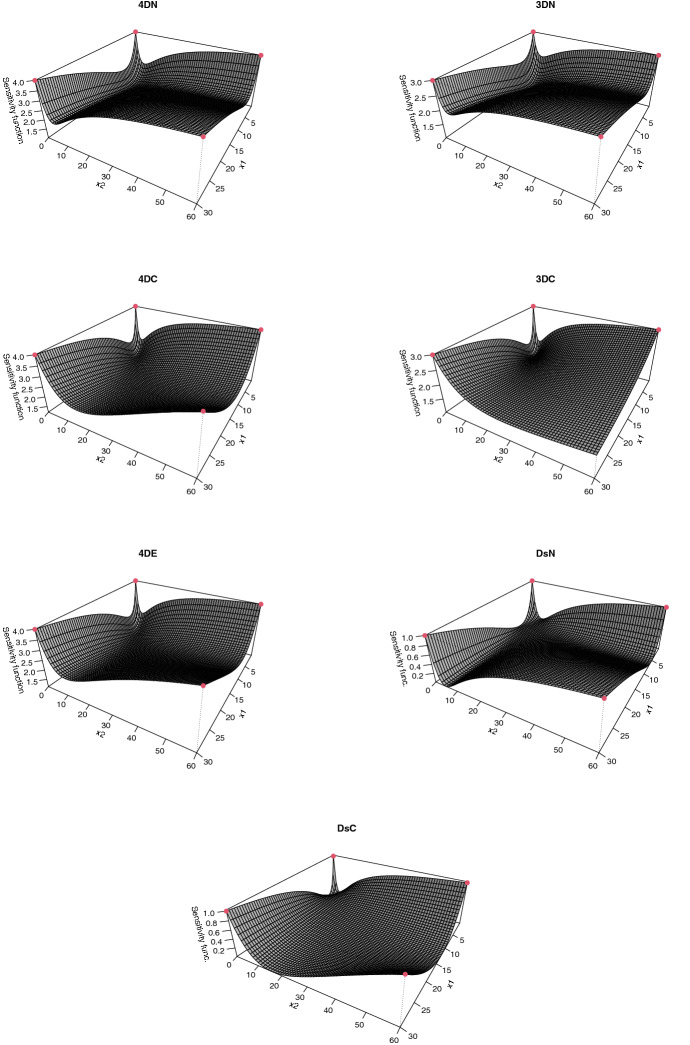
Plot of sensitivity functions for *D*- and $D_s$-optimal designs

**Fig. 5:**
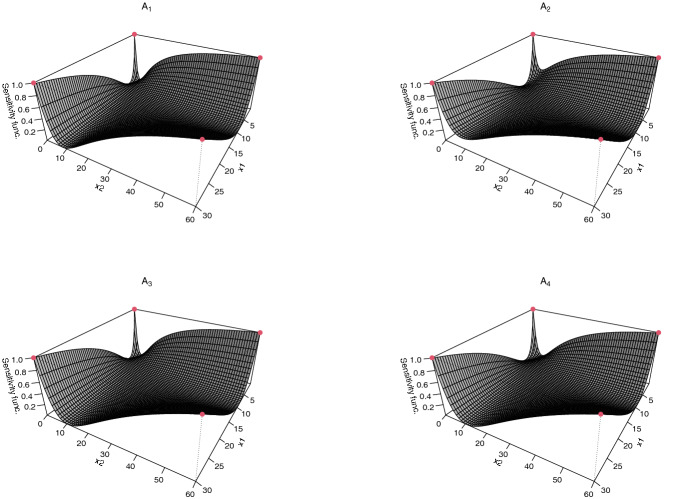
Plot of sensitivity functions for *T*-optimal designs

According to the estimated parameters in Table 2, $\mathrm{IC}_{50}$ (see Appendix 1) is equal to $\mathrm{IC}_{50}=6.638$ for the encompassing model in the log case using (Eq. 20). Determination of the reversible inhibition modality of a compound is of high importance in biopharmaceutical studies to observe whether an inhibitor may be detached from the enzyme complex, after the trace of its effect as a ligand is perceived (basically to reduce the side effects of using a ligand). For this purpose, different $\%$ inhibition, defined in (Eq. 19) below, needs to be determined. Together with different substrate concentrations, concentrations of both must simultaneously vary to determine the effect of these changes (forming a 96-well plate or other typical ones) on the reaction rate of the target enzyme. Here using different optimality criteria, when determination of the enzyme type is not possible or hardly possible (i.e., for discrimination purposes), we have computed these compounds (simultaneous concentrations of both the substrate and inhibitor) in an optimal way presented in Table 5. Further, the substrate titration and inhibitor concentration ranges are slightly changed compared to Copelands suggestions [3], Chapter 5, to match with our assumed design region and to make the results of these experimentation more feasible. Therefore, the concentrations of inhibitor relative to $\mathrm{IC}_{50}$ (for the encompassing model) for four different inhibitor concentrations each evaluated in triplicate, are used to form a similar of a 96-well plate format using optimal design $A_3$ to help visualization of concentration–response plots and other similar interpretations for an interested investigator, using the following equation

19 $$\begin{aligned} x_{I}=\mathrm{IC}_{50}\left( \dfrac{E_0[y]}{E_i[y]} -1 \right) , \end{aligned}$$

for the Hill coefficient equal to one (which suggest a well-behaved concentration–response relationship). Table 7 for different $\%$ inhibition (here, 0, 50, 75, and $90\%$ inhibition which have been chosen relative to $\mathrm{IC}_{50}=6.638$ and taking into account the assumed upper bound of $[x_I]_{\max }=60$ in the design region) helps to provide a convenient scheme for simultaneous inhibitor and substrate titration in a 96-well plate plotted next in Fig. 3.

**Table 7:** Concentrations of inhibitor relative to $\mathrm{IC}_{50}=6.638$ for different inhibition levels

$\%$ Inhibition	Fractional activity $(E_i[y]/E_0[y])$	$E_0[y]/E_i[y]$	$x_I$
0	1	1	0
50	0.50	2	$\mathrm{IC}_{50}$
75	0.25	4	$3 \mathrm{IC}_{50}$
90	0.10	10	$9 \mathrm{IC}_{50}$

A similar visualized result, compatible with the information in Table 5, observed from the 96-well plate (Fig. 3, the right one) is that using the optimal designs for discriminating between the enzyme log models ($A_3$ here), one does not need to simultaneously vary multiple pair concentrations for further investigation of velocity equations and curve fitting to the entire dataset. Instead, for example, the suggested design $A_3$ require only two substrate and inhibition titrations which require only one level change in each of substrate and inhibition concentrations (drawn in red thick vertical and horizontal lines, respectively) as opposed to wide titration ranges which are usually used for both concentrations (Fig. 3, the left one) in curve fitting and similar applications. The shading relates to the resulted weights for the design $A_3$ (see Table 5). A similar procedure could be applied to provide 96-well plates for other optimal designs computed in this work for investigators having interest in other calculated designs either for estimation or discrimination (i.e., Tables 3 and 5).

All these findings clearly point out how careful the experimenter needs to be in her/his decisions not only about the models used, but also the error structure and the form and boundaries of the design region. As usual, however, any effort invested in the experimental design pays off, if those choices stay within reasonable ranges.

## Appendix 1: Fractional Activity and $\mathrm{IC}_{50}$ Determination

In drug discovery terminology, at any concentration of inhibitor, the total concentration of enzyme in the sample is, by mass-balance, equal to the sum of the concentration of free enzyme molecules and the concentration of enzyme–inhibitor complex and therefore the fractional activity, the expected reaction rate of the free enzyme over the total enzyme concentration can be defined as $E_i(y)/E_0(y)$ [3]. The fraction of enzyme occupied by the inhibitor, can also be shown by $1-(E_i(y)/E_0(y))$ and the $\%$ inhibition is accordingly equal to $100(1-(E_i(y)/E_0(y)))$. Therefore, both plots of fractional velocity and the same behavior on a semilog plot (same plot on a different scaling and log transformation of data) will be decreasing functions of inhibitor concentrations. Finally the fractional velocity of 0.5, corresponding to $50\%$ inhibition of the target enzyme which is basically referred to as inhibitor concentration at fractional activity of 0.5 determines the $\mathrm{IC}_{50}$ value. These calculations for the encompassing model (by comparing the expected reaction rates of the encompassing model and that of the Michaelis–Menten model at $x_{S}=\theta _{M}$ and using the definition of $\mathrm{IC}_{50}$) results in

20 $$\begin{aligned} \mathrm{IC}_{50}=2\theta _{K}/(2-\lambda ) \end{aligned}$$

[4]. Also for competitive and non-competitive inhibition models $\mathrm{IC}_{50}$ could be driven from Eq. (20) using their respective inhibition constants. These all can suggest the non-negativity of the third parameter, $\theta _{K}$ (of the encompassing model), and similarly those of competitive and non-competitive inhibition models.

## Appendix 2: The Quadratic Design Criterion

Table 8 presents the design based on the quadratic design criterion proposed in [24] for the encompassing model in both the standard and the log case.

**Table 8:** Designs resulted from the quadratic design criterion for the encompassing model

Standard case			Log case		
$x_S$	$x_I$	$\omega$	$x_S$	$x_I$	$\omega$
30.000	0.000	0.243	0.02	0	0.210
3.615	0.000	0.244	30.00	0	0.256
30.000	18.136	0.249	0.02	60	0.234
7.267	7.983	0.264	30.00	60	0.300

## Appendix 3: Equivalence Theorems

### Equivalence of *D* and *G* Extremum Problems

An analogue of the celebrated equivalence theorem [32] which states equivalence of two extremum problems, approximate *D*-optimum and *G*-optimum (Eq. (21)) designs, can be formulated for nonlinear models [33]. Using this useful property, one can check whether a computed design is actually *D*-optimum. A *G*-optimum design minimizes the maximum over ${\mathbf {x}}$ of the sensitivity function and is defined as

21 $$\begin{aligned} d({\mathbf {x}},\xi ,{\bar{\varvec{\theta }}})=f^{T}({\mathbf {x}},{\bar{\varvec{\theta }}}) M^{-1}(\xi ,{\bar{\varvec{\theta }}})f({\mathbf {x}},{\bar{\varvec{\theta }}}). \end{aligned}$$

The equivalence theorem states that the following three conditions are equivalent:

1. Design $$\xi ^{*}$$ maximizes $$\Phi _{D}(\xi ,{\bar{\varvec{\theta }}})$$.
2. Design $$\xi ^{*}$$ minimizes $$\max _{{\mathfrak {X}}} d({\mathbf {x}},\xi ,{\bar{\varvec{\theta }}})$$.
3. $\max _{{\mathfrak {X}}} d({\mathbf {x}},\xi ^{*},{\bar{\varvec{\theta }}})=m$, where *m* is the number of parameters in the model and the maxima occur at the points of support of the optimal design, i.e., $d({\mathbf {x}}_{i}^{*},\xi ^{*},{\bar{\varvec{\theta }}})=m$. Therefore, for any non-optimum design $\xi$,
4. $$\max _{{\mathfrak {X}}} d({\mathbf {x}},\xi ,{\bar{\varvec{\theta }}})>m$$.

For an analogous $D_s$-optimality equivalence theorem one may refer to [23].

### *T*-optimality Equivalence Theorem

Here, we represent the general case of the theorem for *CT*-optimal designs, similar to the results of [34], which works for any value of $\nu$ including $\nu =0$ and $\nu =1$ as

1. a necessary and sufficient condition for a design $\xi _{CT}^{*}$ to be *CT*-optimal is fulfillment of the inequality
  22 $$\begin{aligned} \Psi _{CT}({\mathbf {x}},\xi _{CT}^{*}) \le 1, \quad {\mathbf {x}}\in {\mathfrak {X}}, \end{aligned}$$
  with the sensitivity functions $\Psi _{CT}({\mathbf {x}},\xi )=\left( 1-\nu \right) \dfrac{\Psi _{0}({\mathbf {x}},\xi )}{\Delta _0(\xi )}+\nu \dfrac{\Psi _{1}({\mathbf {x}},\xi )}{\Delta _1(\xi )}$, $\Psi _{0}({\mathbf {x}},\xi )=\left( \eta _0({\bar{\varvec{\theta }}}_0,{\mathbf {x}})- \eta _1({\hat{\varvec{\theta }}}_1,{\mathbf {x}})\right) ^2, \Psi _{1}({\mathbf {x}},\xi )=\left( \eta _1({\bar{\varvec{\theta }}}_1,{\mathbf {x}})- \eta _0({\hat{\varvec{\theta }}}_0,{\mathbf {x}})\right) ^2$;
2. at the points of the optimum design $\Psi _{CT}({\mathbf {x}},\xi _{CT}^{*})$ achieves its upper bound that is $\Psi _{CT}({\mathbf {x}}_{i}^{*},\xi _{CT}^{*})$;
3. for any non-optimum design $\xi$, that is a design for which $\Phi _{CT}(\xi )<\Phi _{CT}(\xi _{CT}^{*})$, $\sup _{{\mathbf {x}}\in {\mathfrak {X}}}\Psi _{CT}({\mathbf {x}},\xi )>1.$

## Appendix 4: Sensitivity Functions

In this part, we present plots of the sensitivity functions, Figs. 4 and 5, corresponding to all resulted approximate designs in the log case, Tables 3 and 5, respectively. These plots reflect a check of the equivalence theorems in Appendix 3.

## Declarations

### Supporting information

EYs research was fully supported and WMs research was partially supported by project grants LIT-2017-4-SEE-001 funded by the Upper Austrian Government, and Austrian Science Fund (FWF): I 3903-N32.
